# Packaging Design and Thermal Characterization of 3D Double-Sided Cooling Automotive SiC Power Modules with Reliable Junction Temperature Sensing

**DOI:** 10.3390/s26144336

**Published:** 2026-07-08

**Authors:** Chunzhen Li, Tianliang Lin, Xinhua Guo, Rongkun Wang, Yuanxi Chen, Siqi Zhou

**Affiliations:** 1 College of Mechanical Engineering and Automation, Huaqiao University, Xiamen 361021, China; 22011080006@stu.hqu.edu.cn (C.L.); ltlkxl@163.com (T.L.); zhousiqi@stu.hqu.edu.cn (S.Z.); 2 Fujian Key Laboratory of Green Intelligent Drive and Transmission for Mobile Machinery, Xiamen 361021, China; 3 Machinery Industry Key Laboratory of Green Electromechanical Hydraulic Drive and Transmission Technology for Construction Machinery, Huaqiao University, Xiamen 361021, China; 4 College of Information Science and Engineering, Huaqiao University, Xiamen 361021, China; wangrongkun@hqu.edu.cn (R.W.); yuanxi.chen@hqu.edu.cn (Y.C.); 5 Xiamen Key Laboratory of Specialized Integrated Circuits and Power Semiconductor Systems, Xiamen 361021, China; 6 Fujian Provincial University Engineering Research Center for High-Efficiency, High-Density Electromagnetic Power and Systems, Xiamen 361021, China

**Keywords:** double-sided cooling, junction temperature sensing, SiC power module, thermal analysis, vertical chip stacking

## Abstract

Accurate junction temperature ($T_{j}$) sensing is essential for the reliability of silicon carbide (SiC) power modules in electric vehicles. Nonetheless, the physical separation and consequent thermal signal delay between sensing elements and chips pose significant challenges to precise junction temperature monitoring. To solve this issue, an embedded temperature sensing structure integrated into the designed double-sided cooling (DSC) SiC power module is proposed, which leverages 3D vertical interconnects to enhance temperature observability. The customized design of a copper spacer serves as the primary heat dissipation path and electrical connection between the upper and lower chips in the same location. A compact thermal resistance network and 3D finite-element simulations are developed to reveal the vertical thermal coupling between the spacers and the chips, enabling accurate junction temperature estimation from spacer temperature. The proposed concept is experimentally validated on a fabricated prototype using embedded K-type thermocouples and an IR camera under power cycling conditions. The measured temperature differences between the copper spacers and the junction temperature are maintained within approximately 0.5–2 °C under the tested operating range. This approach provides a potential application in real-time condition monitoring and thermal management in high-power-density electric drives.

## 1. Introduction

Power electronic converters are moving towards high efficiency, high power density and high reliability [1]. As the core component of converters, power modules require advanced packaging technologies to improve system performance [2]. In recent years, silicon carbide (SiC) power devices have been gradually replacing traditional silicon (Si) power devices in power converters, particularly in electric vehicle (EV) inverters, due to their excellent characteristics such as high switching speed, high power density and high temperature application potential [3,4]. Consequently, the operational and health status of power modules determines the reliability and performance of the entire system, thereby ensuring safe and efficient operation [5]. Junction temperature ($T_{j}$) is a pivotal indicator of power modules. Accurate $T_{j}$ monitoring is essential for preserving the modules’ stability and provides a critical parameter for life prediction and health management [6].

The $T_{j}$ testing methods for power modules are divided into two aspects: $T_{j}$ estimation [7] and $T_{j}$ sensing. The $T_{j}$ estimation methods are the thermal-sensitive electrical parameter (TSEP) method [8] and the thermal network model method [9]. $T_{j}$ sensing methods are the physical contact and noncontact temperature sensing methods [10].

The TSEP method estimates $T_{j}$ indirectly by exploiting the relationship between the electrical parameters of the device and the junction temperature, which is suitable for real-time temperature measurement, but it is easily affected by electromagnetic interference, equipment noise and other factors. The thermal network model method conducts numerical and reduced-order thermal models. The numerical model method mainly uses simulation software such as the finite element analysis (FEA) method and the finite difference method (FDM), but it has the disadvantages of long simulation time and inability to detect the junction temperature in real time [11].

Physical contact methods include negative temperature coefficient (NTC) thermistors [12], Fiber Bragg Grating (FBG) temperature sensing technology [13], on-chip temperature sensors [14] and in situ sensors [15]. NTC thermistors are widely used to prevent power modules from overheating owing to their low cost and broad universality, yet they are intrinsically constrained by a slow transient response. FBG sensor method can offer real-time $T_{j}$ monitoring, but it is currently expensive, necessitates on-chip installation and additional terminals, and is mainly used in IGBT. The on-chip integrated temperature sensor method achieves customization by incorporating temperature sensors into the chip during the chip design and manufacturing stages, such as RC-IGBT. This introduces additional manufacturing costs and limits universality, and the impact of chip aging requires further verification. Ke et al. first utilized micro-nano fabrication technologies to develop an in situ $T_{j}$ sensor within the power module, enabling online temperature monitoring and enhancing universality. The manufacturing process of the sensor was integrated with the existing module packaging workflow [16]. However, since the in situ sensor was attached to the chip solder to measure the bottom temperature of the chip, the accuracy of this method is compromised by material aging. Consequently, accelerated aging experiments must be conducted to further validate the feasibility of this approach. The noncontact temperature method typically uses an infrared thermal imaging camera to measure $T_{j}$, but this method requires opening the module cover and removing insulating silicone. To increase temperature measurement accuracy, the module needs to be coated in black paint. Therefore, the IR camera is primarily restricted to laboratory environments [17].

Traditional single-sided cooling (SSC) packaging architecture is based on wire-bonding electrical interconnection. Monitoring $T_{j}$ of SSC power modules typically involves indirect temperature sensing or is restricted by module structural limitations. The commercial approach is to use NTC thermistors to indirectly obtain the $T_{j}$. However, the NTC thermistor is relatively far from the chip. To enhance the thermal response and temperature sensing accuracy, it is necessary to take the NTC closer to $T_{j}$ [18]. While recent studies have explored data-driven approaches for junction temperature estimation [18,19,20], these methods rely on complex algorithms and training data and may not fully address the fundamental challenge of physical signal delay caused by sensor-chip separation.

Double-sided cooling (DSC) offers several advantages over SSC packaging, such as reduced junction-to-case thermal resistance ($R_{th\_jc}$), lower module parasitic inductance ($L_{module}$) and a more compact current loop structure [21]. These advantages contribute to increasing power density and efficiency of power converters. DSC structures connect the top and bottom direct bonding copper (DBC) to create two parallel cooling paths, achieving a low thermal resistance and low inductance power loop to improve the electro-thermal performance of the power module [22]. Although the compact 3D stacked DSC modules pose a challenge for measuring chip surface temperature directly, DSC technology improves heat dissipation by adding additional heat pathways. Replacing most of the bonding wires with metal connection blocks, DSC provides electrical interconnection and heat transfer through electrical contact areas larger than those of bonding wires and allows for integration of temperature sensors in these connection blocks [23].

Recent advances in DSC power module packaging have primarily targeted electrical and thermal improvements, whereas $T_{j}$ monitoring remains an area with limited investigation. For high-temperature performance of DSC SiC power modules, Wu et al. used a hermetic conformal coating to design modules for high-temperature operation and used infrared imaging to observe the operating condition at 250 °C [24]. Zhu et al. designed a compact spatially symmetric double-sided embedded packaging and studied its thermal performance at junction temperature up to 500 °C [25]. For the DSC structure design, Yang et al. presented an interleaved planar package that improved the parallel current balance among multiple chips and achieved an excellent uniform thermal distribution [26]. The vertical stacking structures and 3D power module provided a compact layout, which is conducive to enhancing the SiC power device performance. This can be realized through vertical component connection and 3D layout to reduce current paths and mitigate mutual inductance [27]. Huang et al. developed a low-temperature co-fired ceramic (LTCC) as an interposer to facilitate current path and thermal distribution, utilizing nickel-plated copper balls in place of bonding wires for chip interconnection to realize a DSC stacked wire-bondless power module package [28]. Seung et al. demonstrated a method to reduce the thermal stress on the chip by optimizing the spacer structure of the DSC structure and analyzed the operational reliability of the module by indirectly calculating junction temperature derived from the module’s k coefficient through the power cycling tests [29]. Overall, the packaging design for 3D layout and DSC structures mainly focuses on enhancing the electro-thermal performance of SiC power modules, overlooking the estimation and monitoring of junction temperature in such DSC power modules.

To address the gap between high-performance packaging design and reliable $T_{j}$ sensing in DSC SiC power modules, this paper presents an embedded temperature sensing strategy that is integrated directly into the packaging design phase. A 3D vertical chip-stacked DSC SiC power module is developed. Instead of the traditional electrical interconnection wire bonding method, this approach utilizes the internal copper spacers, which serve as both electrical vertical chip interconnects and topside heat dissipation pathways, as temperature sensing proxies. In contrast to previous DSC packaging studies that mainly focused on reducing thermal resistance and parasitic inductance, the proposed approach co-designs the 3D structure and sensing scheme to enhance junction-temperature observability without compromising electro-thermal performance. The vertical thermal coupling between the chips and spacers is analyzed through a compact thermal resistance network and 3D simulations, and the embedded sensing concept is validated experimentally on a fabricated prototype.

This paper focuses on the SiC MOSFET power modules with a DSC structure based on vertical chip stacking. Section 2 elaborates on the design concept of the proposed power module, and Section 3 analyzes thermal performance through steady-state and transient thermal simulation of the proposed modules. Section 4 describes the manufacturing process of the module and the junction temperature test verification of the module. Section 5 presents the summary and the future work.

## 2. Proposed 3D Chip Stacking DSC Power Module

### 2.1. Profile of the 3D Chip Stacking Power Module

**Figure 1:**
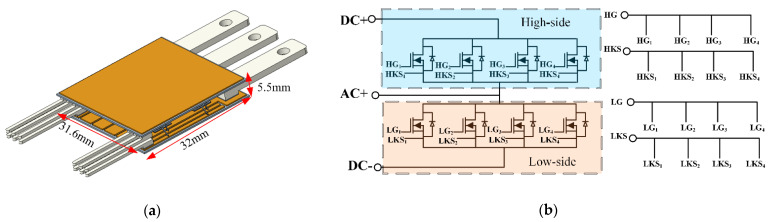
Proposed 3D DSC power modules (**a**) Layout; (**b**) Half-bridge equivalent circuit.

**Figure 2:**
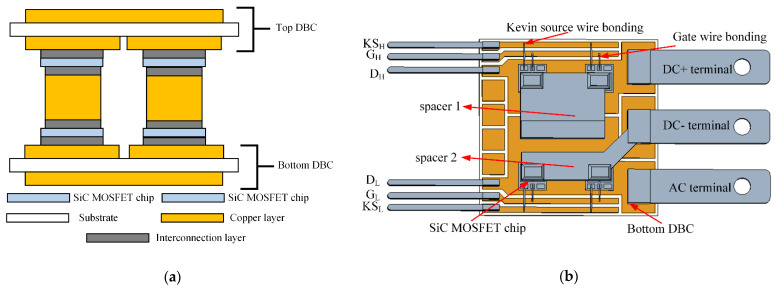
Interconnection structure of the proposed module. (**a**) Cross-sectional view of proposed 3D vertical chip stacking structure; (**b**) stacked view of the bottom DBC.

This paper designs a SiC power module with multiple chips in parallel and a DSC structure. The overall structure is shown in Figure 1a, and the overall dimensions of the module are 32.0 mm × 31.6 mm × 5.5 mm. The half-bridge topology of the 1200 V/140 A silicon carbide MOSFET chip is selected, as shown in Figure 1b. Figure 2a shows the cross-section of the proposed module, and Figure 2b illustrates its internal structure and DBC layout. The module integrates eight SiC MOSFET dies, with four chips connected in parallel to form each bridge arm. The SiC chips are connected to the DBC through the solder layer and spacers. The power loop electrical connections are achieved by copper spacers instead of bonding wires.

The proposed module is composed of multiple layers of material. From top to bottom, these layers are the top DBC, top chips interconnection layer, top chips, top spacer interconnection layer, spacer, bottom spacer interconnection layer, bottom chips, bottom chip interconnection layer and bottom DBC, forming a double-sided heat dissipation structure. Among them, the chip interconnection layer is the interconnection between the chip and DBC, and the spacer interconnection layer is the connection between the chip source and the spacer. The top and bottom chips are placed at the same position in the vertical direction. The vertical symmetrical layout is achieved using a copper spacer. Based on the half-bridge topology and current flow direction, the copper spacer is designed with two parts. Spacer 1 connects the high-side chips’ source to the AC terminal and the drain of the low-side chips. Spacer 2 connects the source of the low-side chips to the DC terminal. As shown in the bottom DBC layout in Figure 2b, the upper part is the upper bridge arm layout, and the lower part is the lower bridge arm layout. The six signal terminals are respectively located on the upper side ($D_{H}$, $G_{H}$, ${KS}_{H}$) and the lower side ($D_{L}$, $G_{L}$, ${KS}_{L}$) of the DBC, and the top DBC also has the same layout as the bottom DBC, each with six signal terminals placed, as shown in Figure 1a and Figure 2b.

**Figure 3:**
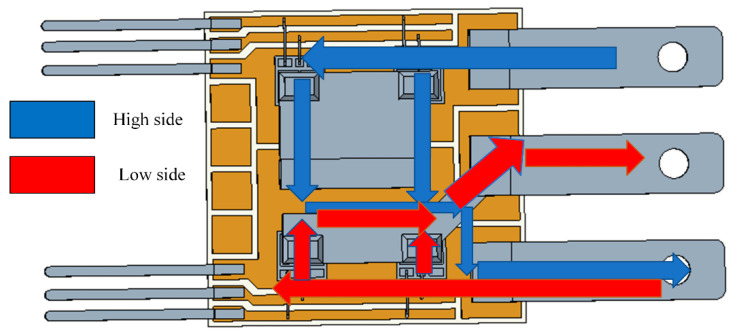
Current path of the proposed module (bottom DBC is used as an example).

**Figure 4:**
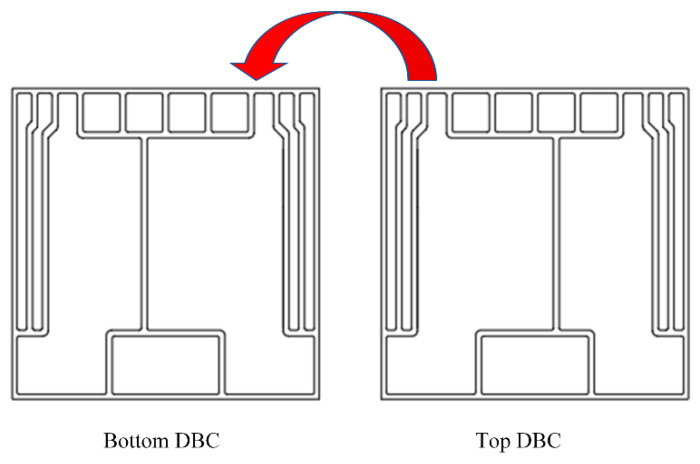
The bottom and top DBC layout.

According to the half-bridge topology in Figure 1b, Figure 3 provides the current flow of the designed power modules. The DC+ terminal is interconnected with the top DBC and bottom DBC, and the chip drain pad is interconnected with the DBCs. The chip source pad is vertically stacked with multiple SiC chips in parallel using a copper spacer, which serves for electrical connection and heat transfer. The chip source pad forms an electrical connection with the AC terminal, which constitutes the upper bridge arm from the DC+ terminal to the AC terminal. The AC terminal is interconnected with the lower chip drain, and the lower chip source is connected to the DC- terminal through a spacer. The current path from the AC terminal to the DC- terminal forms the lower bridge arm. The bonding wire is used to connect the gate and the Kelvin source of the chip for the driving loop. Based on the chip stacking structure and current flow direction, the proposed stacking structure forms a vertical interconnection structure by replacing the chip interconnection bonding wire with spacers, based on the SSC heat dissipation DBC layout. However, it should be noted that when the top DBC and bottom DBC layouts are vertically arranged, there will be some differences in the DBC position, as shown in Figure 4.

### 2.2. Comparison of SSC and Proposed Power Modules

**Figure 5:**
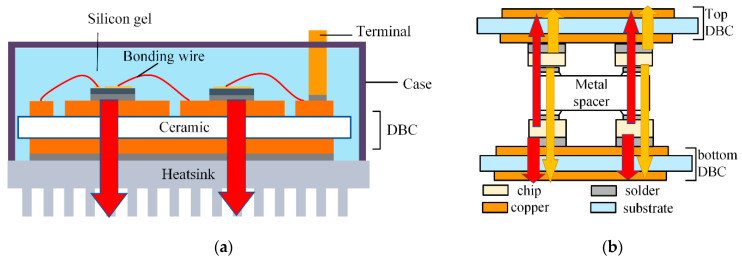
Comparison of thermal structure. (**a**) traditional SSC; (**b**) proposed 3D DSC.

The SSC packaging structure is shown in Figure 5a. The DSC power module in Figure 5b adds a top interconnection path, which provides two heat dissipation paths on the chip topside. According to the half-bridge topology and the current flow direction relationship of multichip parallel, the proposed vertical stacking structure places chips on both the top DBC and the bottom DBC. Through the chip path, the source area of the chips is vertically connected. The chips serve as the main heat source. Taking the bottom chip as an example, there are two heat dissipation paths. One dissipates heat through the solder layer and the bottom DBC. The other dissipates heat through the bottom chip topside, passing through the spacer, top chips and top DBC. To better achieve the vertical interconnection of the chip source, the metal spacer interconnection part is customized, and the interconnection part is treated with grooves to facilitate positioning during the module fabrication.

The thermal dissipation analysis compares the SSC structure and the proposed DSC chip stacking structure in Figure 5. In the SSC structure, heat dissipation occurs primarily through a single path from the chips’ bottom to the DBC, then to the heatsink and the surrounding environment. The bonding wire is used for electrical connection, and the silicone for electrical insulation at the chips’ topside. In contrast, the chip stacking design maintains the planar layout of SSC structures but adds a vertical heat dissipation path, enabling heat to dissipate simultaneously from both the upper and lower chip surfaces. This results in uniform and symmetrical heat dissipation in the vertical direction, given the identical placement and heat generation of upper and lower chips during operation. Subsequently, the thermal simulation analysis of the vertical heat dissipation is detailed in Section 3.

## 3. Thermal Modeling and Analysis of the Proposed 3D Chip Stacking Power Module

### 3.1. Thermal Resistance of the Proposed DSC Power Module

To theoretically quantify the thermal performance of the proposed 3D chip stacking and DSC structure, a thermal resistance network model of the module is established in this paper. In traditional SSC modules, heat is often generated in the chips and dissipated through a downward thermal path from the chip to the heat sink, and its equivalent thermal resistance $R_{th,i}$ can be expressed as:

(1) $$R_{th,i}=\frac{L_{i}}{k_{i}A_{i}}$$

where $R_{th,x}$, $k_{x}$, $L_{x}$ and $A_{x}$ are the thermal resistance, thermal conductivity, thickness and equivalent thermal conduction area of i-th layer, respectively.

For the DSC power modules, the junction-to-case thermal resistance $R_{th}$ can be proposed as follows [30]:

(2) $$R_{th}=\frac{R_{thjct}\cdot R_{thjcb}}{R_{thjct}+R_{thjcb}}$$

where $R_{thjct}$ and $R_{thjcb}$ are junction-to-case thermal resistances of thermal paths on the top side and bottom side.

For the proposed interconnect architecture, the $R_{spacer}$ and ${∆T}_{spacer}$ of the spacer that interconnects the upper and lower chips can be expressed as follows [30]:

(3) $$R_{spacer}=\frac{L_{spacer}}{k_{spacer}\cdot A_{spacer}}$$

(4) $${∆T}_{spacer}=T_{spacer\_top}-T_{spacer\_bom}$$

According to the heat flow direction indicated in Figure 5, taking the bottom chip as an example, compared with the SSC structure, there is an increase in heat dissipation from the top of the bottom chip and the top DBC vertically. However, since the chips at the same position on the top DBC are also conducting when the bottom chip turns on, and the current passing through is similar in magnitude. Therefore, $P_{top}=P_{bot}=P$. Under the condition of double-sided heat dissipation, both the top and bottom DBC are in the same temperature environment, that is, $T_{c\_top}=T_{{c\_}_{bot}}=T_{a}$. Since the proposed layout is a symmetrical structure, the upper and lower chips use the same material and thickness. The temperature difference between the upper and lower chip junction temperature and the case temperature is small, that is $R_{top}=R_{bot}=R_{1}$. The smaller part in the direction perpendicular to the chip interconnection can be set as an isothermal surface. The temperature of junction-to-case can be expressed as:

(5) $$T_{j}=T_{a}+PR_{1}$$

$R_{1}$ represents the thermal resistance from the junction to the chip drain through DBC to the ambient, which is the same as the thermal resistance of the SSC structure. Due to the small ${∆T}_{spacer}$ in the vertical direction. When $T_{j1}=T_{j2}$, it is equivalent to the copper spacer being on an isothermal surface in the vertical direction of the chip source interconnection, as shown in the spacer 2 structure. As the heat dissipation components at the top of the chip, the thermal models of spacer 1 and spacer 2 are analyzed in Equations (6)–(16).

(6) $$P_{1}=\frac{T_{j1}-T_{a,bot}}{R_{1,bot}}+\frac{T_{j1}-T_{s,bot}}{R_{2,bot}}$$

(7) $$P_{2}=\frac{T_{j2}-T_{a,top}}{R_{1,top}}+\frac{T_{j2}-T_{s,top}}{R_{2,top}}$$

where $T_{j1},\;T_{a,bot}$, $T_{s,bot}$, $T_{j2},\;T_{a,top}$, $T_{s,top}$ are the junction temperatures of the bottom and top chips, the ambient temperature and the temperature of the connection layer between the chip and the copper spacer, respectively. $R_{1,bot}$, $R_{2,bot}$, $R_{1,top}$ and $R_{2,top}$ are the thermal resistance between the bottom and top chip junctions and the ambient, as well as the thermal resistance of the contact part between the chip junction and the copper spacer, respectively.

According to the structural symmetry relationship of the module, Equations (6) and (7) can be expressed as Equation (8).

(8) $$P=\frac{T_{j}-T_{a}}{R_{1}}+\frac{T_{j}-T_{s}}{R_{2}}$$

The heat flow distribution on the surface of the spacer in contact with the chip source can be divided into the heat flow from the chip, which flows into the external environment in the vertical and horizontal directions through the solder layer.

(9) $$\frac{T_{j}-T_{s}}{R_{2}}=\frac{T_{s}-T_{cu}}{R_{solder}}+\frac{T_{s}-T_{a}}{R_{3}}$$

where $R_{solder}$ represents the solder thermal resistance when the chip source contacts the copper spacer, and $R_{3}$ represents the lateral thermal resistance from the connection position between the chip source and the copper spacer to the ambient.

Due to the small temperature difference between the copper spacer and the $T_{j}$ in the vertical direction, the heat flow cannot dissipate in the vertical direction. Assuming $T_{s}=T_{cu}$, Equation (9) can be concluded from Equations (10)–(12).

(10) $$\frac{T_{j}-T_{s}}{R_{2}}=\frac{T_{s}-T_{a}}{R_{3}}$$

(11) $$R_{3}(T_{j}-T_{s})=R_{2}(T_{s}-T_{a})$$

(12) $$T_{s}=\frac{R_{3}T_{j}+R_{2}T_{a}}{R_{3}+R_{2}}$$

Then, from (8), we can get:

(13) $$P=\frac{T_{j}-T_{a}}{R_{1}}+\frac{1}{R_{2}}(T_{j}-\frac{R_{3}T_{j}+R_{2}T_{a}}{R_{2}+R_{3}})$$

This is written as

(14) $$\frac{1}{R_{2}}\frac{\left(R_{2}+R_{3}\right)T_{j}-R_{3}T_{j}+R_{2}T_{a}}{R_{2}+R_{3}}=\frac{1}{R_{2}}\frac{R_{2}(T_{j}-T_{a})}{R_{2}+R_{3}}=\frac{T_{j}-T_{a}}{R_{2}+R_{3}}$$

(15) $$P=(T_{j}-T_{a})(\frac{1}{R_{1}}+\frac{1}{R_{2}+R_{3}})$$

Thus, the total thermal resistance $R_{total}$ can be concluded as follows:

(16) $$R_{total}=\frac{1}{\frac{1}{R_{1}}+\frac{1}{R_{2}+R_{3}}}$$

This section analyzes the thermal resistance of the spacer in the proposed module under chip heating conditions. It can be observed that spacer 1 introduces a lateral heat dissipation path in addition to the vertical parallel connection. Heat first flows from the source of the chip through the solder layer to the copper spacer. Due to the small temperature difference in the vertical direction of the copper spacer, an isothermal surface appears. The lateral resistance $R_{3}$ reduces the total thermal resistance, thereby reducing $T_{j}$. Due to the lower lateral heat dissipation paths of spacer 2, there is heat accumulation in the vertical direction. To further verify the accuracy of the established thermal resistance model, Section 3.2 analyzes the temperature distribution of the module under steady-state conditions.

### 3.2. Steady-State Thermal Simulation

**Figure 6:**
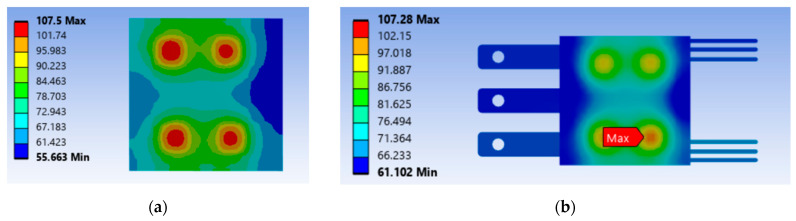
Temperature field distributions of SSC and proposed power module: (**a**) SSC power module; (**b**) proposed DSC power module.

**Figure 7:**
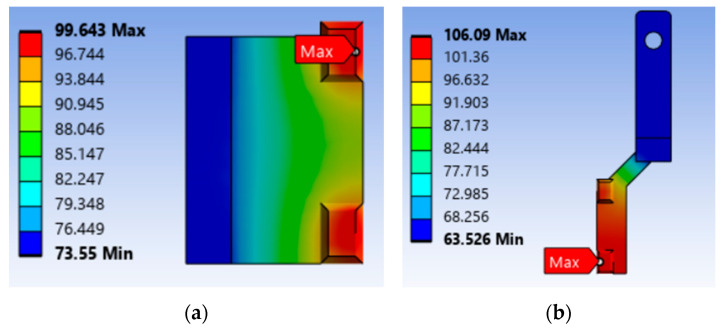
Temperature distributions of the proposed power module chip source connect spacer: (**a**) spacer 1; (**b**) spacer 2.

To better analyze the thermal performance of the proposed stacking structure, the steady-state thermal distribution of the 3D packaging structure was obtained through ANSYS Workbench 2022 R2. Table 1 provides the thickness and material of the proposed 3D modules. Table 2 shows the material properties in the thermal simulation. To verify the heat dissipation performance of the proposed module, the heat dissipation conditions of the same chip layout as the proposed DSC structure in the SSC structure and four chips connected in the proposed DSC structure were compared and analyzed under steady state. The thermal boundary conditions are set as follows: the chip power loss is 60 W, and the top and bottom heat flux at the DBC surface are set at 5000 W/(m^2^·K) to simulate the liquid cooling conditions. In comparison of the thermal characteristics of the proposed structure and the traditional SSC structure, the SSC DBC chooses the same dimension of each bridge arm and uses the Pb_92.5_Sn_5_Ag_2.5_ solder as the chip connection material. Figure 6 shows the steady-state thermal simulation results of the SSC and the proposed DSC structure. Figure 7 provides the thermal distribution of the source connection spacer for high-side and low-side chips.

The heat generation rate of the chip $H_{heat}$ is calculated as follows:

(17) $$H_{heat}=\frac{P_{chip}}{A_{chip}}$$

where $P_{chip}$ denotes the power loss for the chip, $A_{chip}$ is the volume of the chip.

As shown in Figure 6, the thermal distribution diagrams of the SSC structure and the proposed DSC structure were presented in Figure 6a and Figure 6b, respectively. Under the same heat generation condition, the proposed structure shows the same maximum junction temperature as the SSC structure.

Figure 7 shows the thermal distribution of the copper spacer connecting the two chips in the proposed structure. It can be observed that the heat dissipation effect of spacer 1 is better than that of spacer 2, and the highest temperature is located at the contact area with the chip source. This position corresponds to the hottest point on the copper spacer. Moreover, a comparison of the temperature distributions in Figure 6b and Figure 7b shows that the maximum temperature of spacer 2 is close to that of the entire module, and the temperature difference is only 1.19 °C. The heat accumulation of spacer 2 was more obvious than that of spacer 1, resulting in a maximum temperature difference of 6.447 °C between the two spacers.

**Figure 8:**
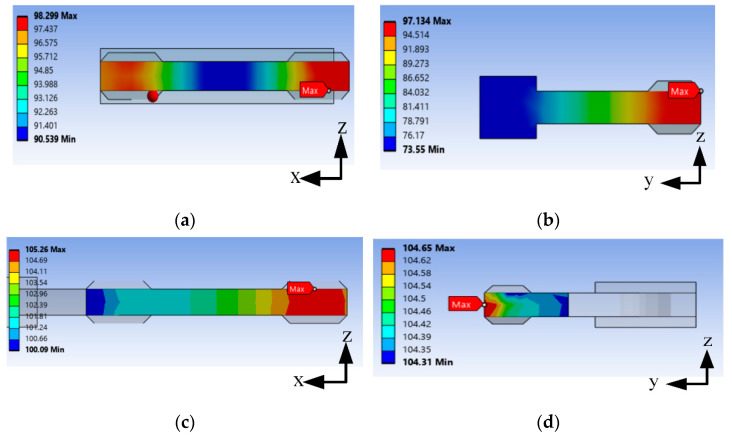
Temperature distributions of the proposed power module chip source connect spacer: (**a**) Spacer 1, X-Z; (**b**) Spacer 1, Y-Z; (**c**) Spacer 2, X-Z; (**d**) Spacer 2, Y-Z.

To further analyze the temperature distribution of the copper spacers, Figure 8 presents the specific thermal distribution diagrams of spacer 1 and spacer 2 in the X-Z and Y-Z directions. In the Y-Z direction, it can be found that the heat accumulation near the chip position along the Z-axis direction cannot be dissipated in the X- and Y- directions. Therefore, thermal equilibrium was established in the vertical direction where the spacer is connected to the chip source, resulting in a higher temperature and a closer position to the chip. According to Figure 8c,d, in the X- and Y- directions, the temperature decreases as the distance from the heat source increases. Spacer 2 has an additional heat dissipation area for the chip close to the DC- terminal, which leads to a temperature decrease in the chip close to the signal terminal. Spacer 1 achieves a higher temperature on both sides and a lower temperature in the middle due to the balanced heat distribution between the chips. Consequently, heat is dissipated towards the middle.

**Figure 9:**
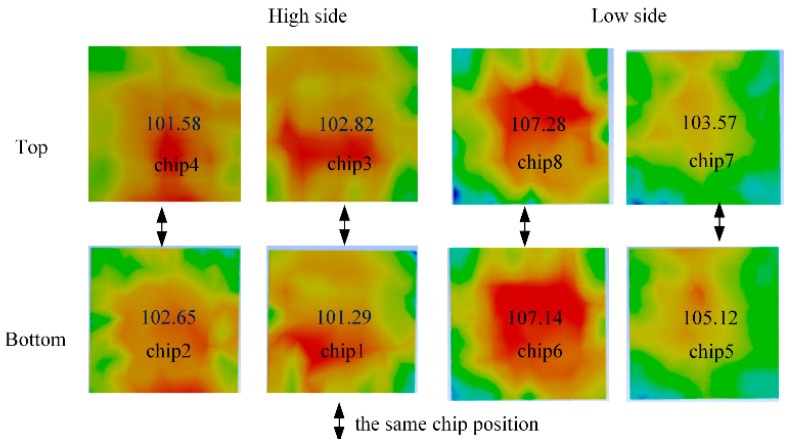
Temperature distributions of each chip in the proposed power module.

Figure 9 illustrates the temperature distribution of each SiC MOSFET chip; Table 3 shows the temperature difference between chips in the vertical position. It can be found that chips at the same vertical position have a low temperature difference, which indicates the 3D chip stacked structures have thermal uniformity. Therefore, due to the low temperature difference in the vertical direction at the same chip position, the heat transfer from the vertical interconnection of the upper and lower chip sources cannot be discharged in time, and the temperature difference between this area and the junction temperature is closely related.

Comparison of simulation results on thermal performance between SSC and stacked DSC modules in the steady state reveals that the stacked DSC modules have the same $T_{j}$ as SSC structures in spacer 2, but DSC structures used spacer 1, which enhances heat dissipation by adding another lateral heat dissipation path to reduce the $T_{j}$.

### 3.3. Power Cycling Thermal Simulation

To better analyze the heat dissipation performance of the proposed stacking structure, the transient thermal distribution of the 3D packaging structure was obtained through ANSYS Workbench. Power cycling simulations were conducted under two conditions —power cycling second (${PC}_{sec}$) and power cycling minute (${PC}_{min}$) to evaluate the junction temperature. The simulation was performed with the DC current of 30 A and 40 A applied to the power modules, and an equivalent heat transfer coefficient $h=100\;W/(m^{2}\cdot K)$ to simulate air cooling as the thermal boundary conditions.

To verify the heat dissipation performance of the proposed module, the thermal behavior of high-side and low-side chips under the stacked DSC structures was compared and analyzed. In DC power cycling mode, the power losses during the heating phase are only conduction losses in SiC MOSFETs, which are calculated in (18) [31]:

(18) $$P_{cond}(T_{j})=V_{ds}(T_{j})\times I_{C}$$

**Figure 10:**
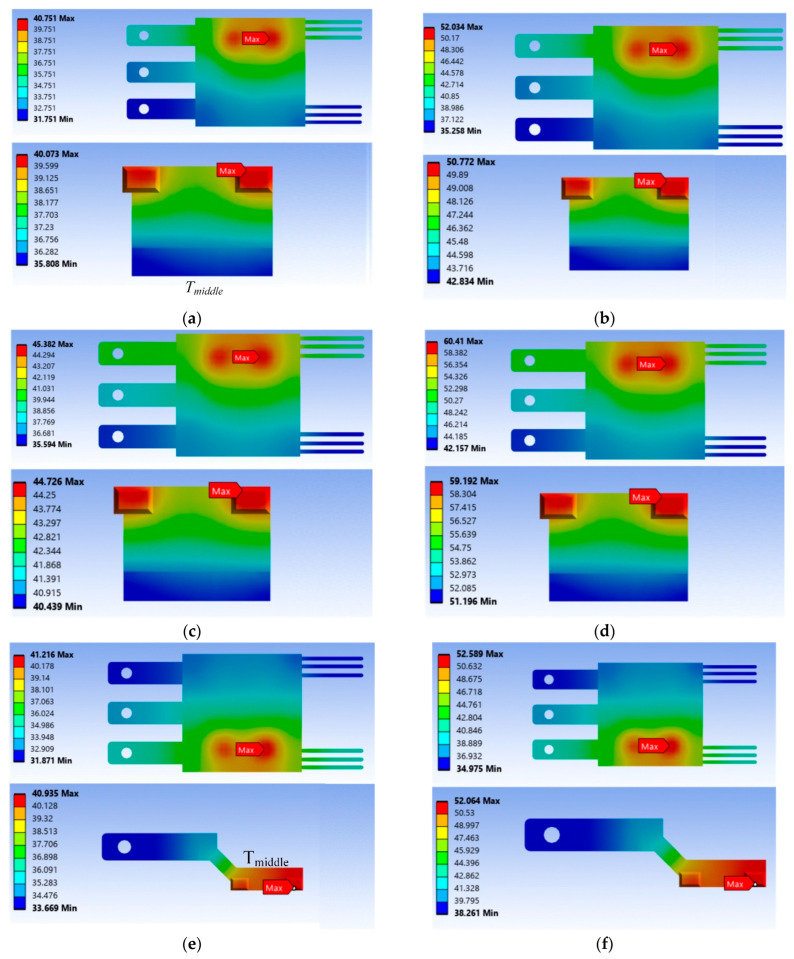

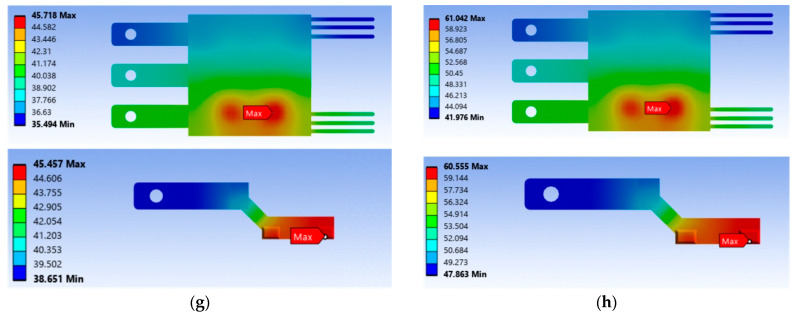
Temperature field distributions of the 3D stack power modules with different chips in parallel: (**a**) ${PC}_{sec}$, 30 A upper arm; (**b**) ${PC}_{sec}$, 40 A upper arm; (**c**) ${PC}_{min}$, 30 A upper arm; (**d**) ${PC}_{min}$, 40 A upper arm; (**e**) ${PC}_{sec}$, 30 A lower arm; (**f**) ${PC}_{sec}$, 40 A lower arm; (**g**) ${PC}_{min}$, 30 A lower arm; (**h**) ${PC}_{min}$, 40 A lower arm.

Transient thermal simulation was conducted under the conditions of ${PC}_{min}$ ($T_{on}=120\;s$ and $T_{off}=160\;s$) and ${PC}_{min}$ ($T_{on}=3\;s$ and $T_{off}=3\;s$) to examine the temperature distribution of the upper and lower bridge arm chips under 30 A and 40 A DC current. According to Equation (18), power losses of 5 W and 9 W are applied to the power module at 30 A and 40 A, respectively, as detailed in Section 4. Figure 10 presents the maximum temperature of the upper and lower bridge arm at both current levels. For the second-level cycle, the maximum junction temperature was recorded at the 15th cycle. For the minute-level cycle, the junction temperature at t=120 s was taken as the reference. The temperature distribution of the copper spacers in the upper and lower bridge arms and the junction temperature are also obtained in Figure 10.

Figure 10 shows the temperature distribution of lower and upper bridge arm chips and spacer 1 under 30 A and 40 A conditions of both ${PC}_{sec}$ and ${PC}_{min}$, while Figure 10e–h show the thermal distribution of lower bridge arm chips and spacer 2. The simulation results indicate that the maximum $T_{j}$ occurs in the chip area close to signal terminals. The temperature distribution of the interconnected area on both sides of the upper bridge arm spacer 1 was uniform, and the temperature gradient in the spacer 1 area that was in close contact with the chip source was gradual. $T_{middle}$ is defined as the minimum temperature inside Spacer 1, observed at the location farthest from the chip heat source. This is shown in Figure 10a. The DC current was increased from 30 A to 40 A; both the overall temperature of the power module and that of spacer 1 rose significantly. Meanwhile, the temperature difference between the maximum temperature and the $T_{middle}$ in spacer 1 had expanded significantly, increasing from 4.264 °C to 7.938 °C, and the heat dissipation performance became more obvious. Due to structural differences, heat accumulation occurred in the vertical interconnection area between spacer 2 and the lower bridge chip, and the heat dissipation capacity of the lower bridge was worse than that of spacer on the upper bridge. Under high current, the heat accumulation in spacer 2 intensifies, hindering the uniform thermal diffusion and efficient heat dissipation from the lower bridge chip.

Based on the steady-state and transient thermal simulation results, the design of spacer1 and spacer2 and the vertical heat path between the chips can provide a more uniform temperature distribution close to the junction temperature, thereby enabling an embedded sensing approach for junction temperature estimation.

## 4. Experimental Verification of the Proposed DSC Structure and $T_{j}$ Monitoring Method

### 4.1. Power Module Prototype Assembly

**Figure 11:**
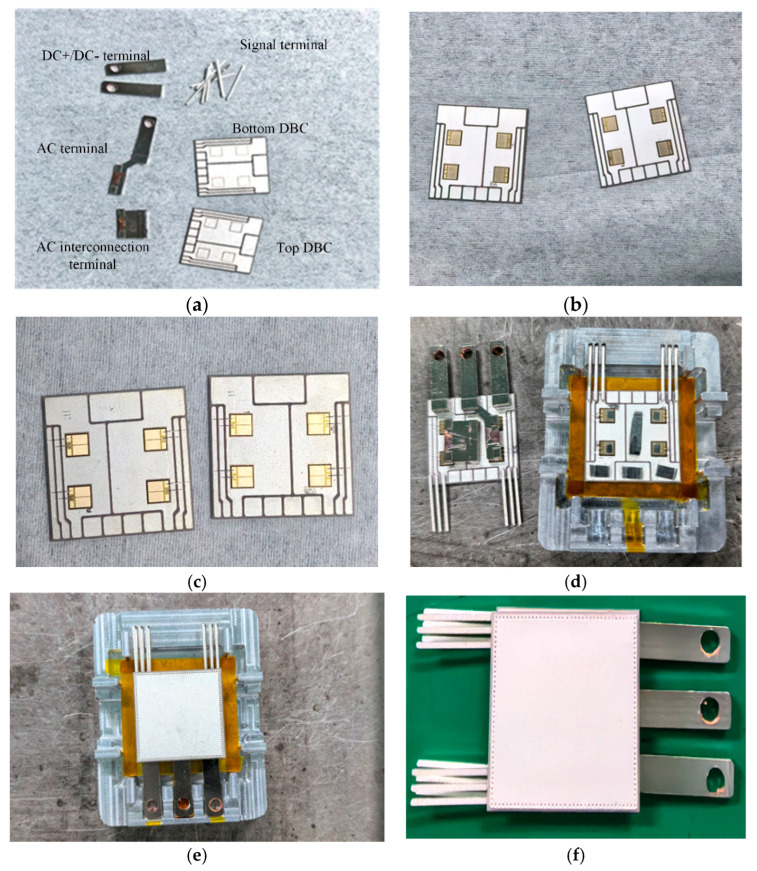
The process of power module assembly: (**a**) main part for the assembly of the proposed DSC power module; (**b**) die attachment; (**c**) wire bonding; (**d**) signal and power terminal attachment, source spacer attachment by Pb_92.5_Sn_5_Ag_2.5_; (**e**) Top view of the final assembly by jig; (**f**) Prototype.

To further validate the proposed 3D vertical packaging design, a prototype of the 3D SiC power module was fabricated. Considering the interconnection relationships among the components within the 3D structure, multiple reflow soldering processes were employed, and the influence of the soldering temperature on the interconnection effectiveness of the solders was taken into account. The fabrication process of the prototype is shown in Figure 11. The components and specifications of the proposed 3D stacked SiC power modules are listed in Table 4.

The manufacturing process of the power module prototype is as follows:

(1) The top DBC and bottom DBC were interconnected with the SiC MOSFET chips. The positions where the SiC chips were to be placed were marked through the etching process to facilitate the placement of preformed high-temperature solder sheets. The high-temperature solder sheets were Pb_92.5_Sn_5_Ag_2.5_ and were soldered in a vacuum welding furnace.

(2) Wire bonding. After the SiC MOSFET chips were soldered in the DBC, the gate and source bonding wires were connected by the wire bonding machine.

(3) The signal terminals were interconnected with the DBC. The top DBC and bottom DBC were vacuum soldered through preformed high-temperature solder Pb_92.5_Sn_5_Ag_2.5_.

(4) The power terminals were interconnected with the DBC. The bottom DBC was chosen to complete the interconnection. The preformed high-temperature solder Pb_92.5_Sn_5_Ag_2.5_ was placed on the DBC together with the DC+ and AC terminals and fixed through a fixture for vacuum soldering.

(5) The DC- terminal, AC interconnection terminal and the source of the bottom chip were interconnected. Cut the preformed Pb_92.5_Sn_5_Ag_2.5_ solder sheets according to the source area of the chip, fixed them with fixtures and then performed vacuum soldering.

(6) Top and bottom DBC package interconnection. After (5), the preformed low-temperature SAC305 solder sheets were placed on the other side of the AC interconnect terminal and the DC-terminal with the bottom DBC. The top DBC was placed on assembly through a fixture, followed by vacuum reflow soldering.

### 4.2. Experimental Verification of the $T_{j}$

In this section, the effectiveness of the junction temperature testing methods by thermal simulation was verified. An experimental platform for junction temperature testing was presented. The junction temperature was tested using a Fotric 225s IR camera. During the packaging process of the prototype, K-type thermocouples were embedded in the copper spacer connected to the SiC chips to monitor the operating temperature of the proposed module, and the junction temperature change process of the module was captured by an IR camera. It is noted that the IR camera measured the surface temperature of the DBC and exposed the part of the module surface, which is close to the junction temperature.

**Figure 12:**
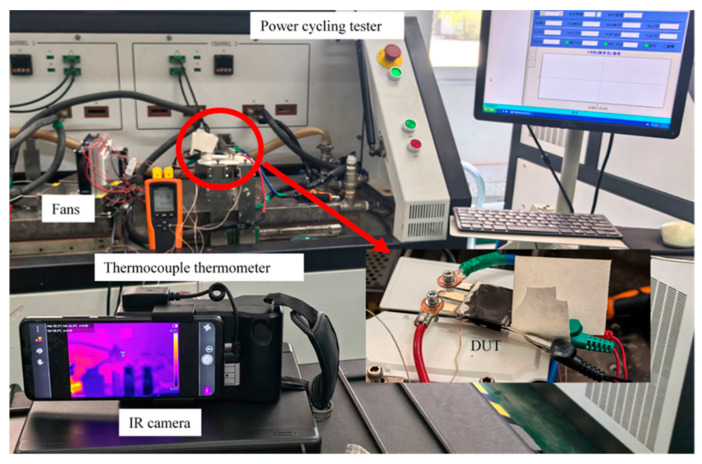
Junction temperature testing platform.

**Figure 13:**
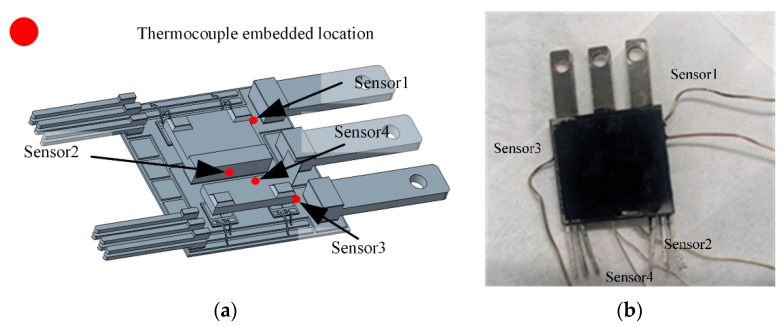
The K-type thermocouple placement position of the module. (**a**) the thermocouple embedded location; (**b**) physical picture (blank paint module).

The experimental platform is shown in Figure 12. A black-painted module was set up in Figure 13b, and a comparative analysis was conducted between the experimental result and the transient thermal simulation result. The assessments were carried out under varying time scales, specifically at minute and second intervals, while subjecting the module to DC currents of 30 A and 40 A. This evaluation was facilitated using a power cycling tester.

The junction temperature was measured by black paint inside the module to increase surface emissivity. To better detect and monitor the temperature fluctuations, black paint was applied to the surface of the upper and lower DBC that exposed to the surrounding environment. The emissivity of the applied paint was determined using a reference sample with known emissivity. The emissivity value was measured as ε=0.95±0.02. The IR camera was carefully focused on the module, and the spatial resolution was selected such that the hot spots associated with the chip locations were clearly resolved. K-type thermocouples were placed at the connection point between the copper spacer and the chip source to monitor the spacer temperature variations. The position of the thermocouples is shown in Figure 13a. An IR camera measured the maximum junction temperature of the power module, which corresponded to the junction temperature. The feasibility of the temperature measurement of the proposed structure was verified by comparing the temperature of the copper spacer collected by the thermocouple with the junction temperature obtained by the IR camera. To characterize the temperature distribution of the module, the device under test (DUT) was deliberately operated without the heatsink and was instead cooled by direct forced-air convection using fans. ${PC}_{sec}$ and ${PC}_{min}$ were employed in the experiment. Due to the inherently limited heat removal capacity of forced-air cooling, non-uniform thermal dissipation across the module was evident. Temperature distributions were acquired under DC current conditions of 30 A and 40 A. For the ${PC}_{sec}$ condition, the thermal transient during the 15th power cycle, specifically within the time interval of 84 s to 90 s, was selected for analysis. For the ${PC}_{min}$ condition, the temperature distribution recorded at 120 s was adopted for evaluation.

### 4.3. Experiment Result and Discussion

**Figure 14:**
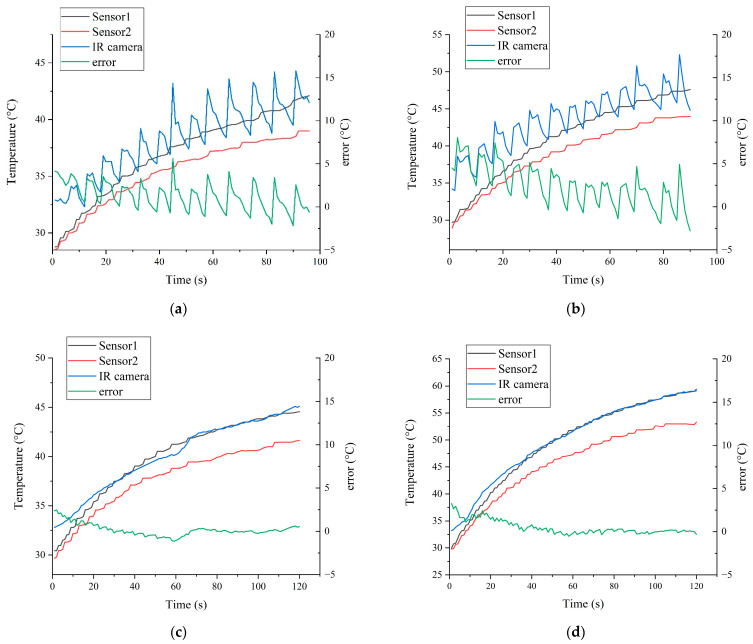
Experimental results of the high-side temperature sensing. (**a**) ${PC}_{sec}$-30 A; (**b**) ${PC}_{sec}$-40 A; (**c**) ${PC}_{min}$-30 A; (**d**) ${PC}_{min}$-40 A. The measured temperature of Sensor1 (black), the measured temperature of Sensor2 (red), the measured temperature by the IR camera (blue) and the data error between the IR camera and Sensor1 (green).

**Figure 15:**
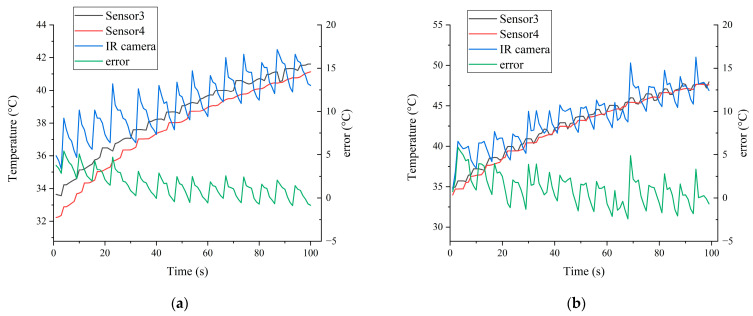

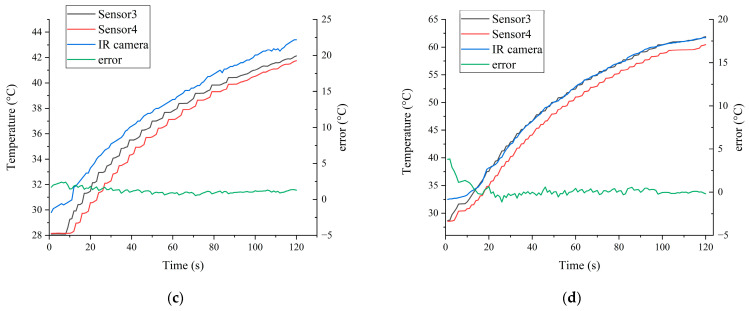
Experimental results of the low-side temperature sensing. (**a**) ${PC}_{sec}$-30 A; (**b**) ${PC}_{sec}$-40 A; (**c**) ${PC}_{min}$-30 A; (**d**) ${PC}_{min}$-40 A. The measured temperature of Sensor3 (black), the measured temperature of Sensor4 (red), the measured temperature by the IR camera (blue) and the data error between the IR camera and Sensor3 (green).

The thermal distribution was obtained through the simulation of second-level cycles and minute-level cycles. The temperature of the copper spacer at different positions was tested by thermocouples under the operation module of the power cycling tester, and the highest temperature obtained after the black paint was sprayed was analyzed. The data curves collected by the power cycling test are shown in Figure 14 and Figure 15. Sensor1 represents the spacer temperature of spacer 1 connected to the upper bridge arm chip in the vertical direction, and Sensor2 represents the spacer temperature of spacer 1 that is farthest from the chip. Sensor3 represents the spacer temperature of spacer 2 connected to the lower bridge arm chip in the vertical direction, and Sensor4 represents the spacer surface temperature of spacer 2 that is farthest from the chip. The IR camera shows the highest temperature collected by the infrared thermal imaging camera. From the power cycling test, the drain-source voltages ($V_{ds}$) measured during operation at 30 A and 40 A are 0.166 V and 0.23 V, from which the power losses imposed on the module are calculated as 5 W and 9 W, respectively.

**Figure 16:**
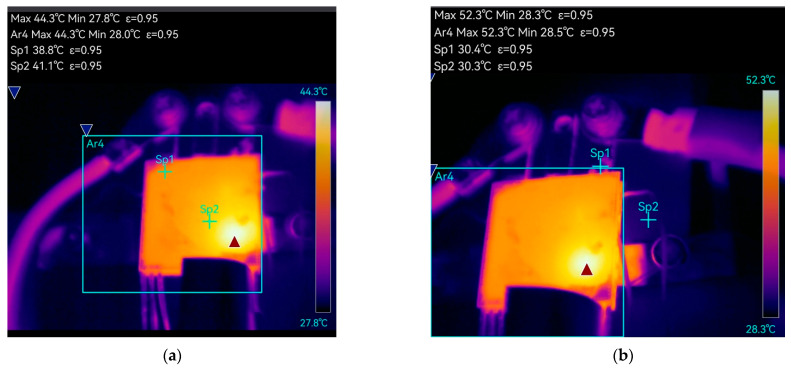

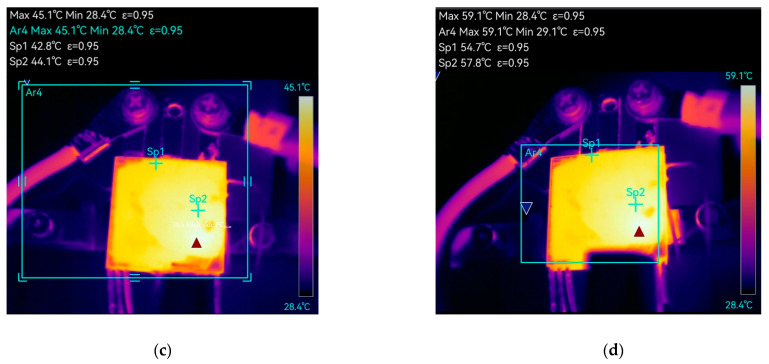
High-side thermal image from IR camera. (**a**) ${PC}_{sec}$-30 A; (**b**) ${PC}_{sec}$-40 A; (**c**) ${PC}_{min}$-30 A; (**d**) ${PC}_{min}$-40 A.

The thermocouple sensor data collected when the upper bridge arm is opened in Figure 14 show that the second-level power cycle can be obtained by observing the temperature change on the DBC surface with the IR camera; see ${PC}_{sec}$ temperature at 30 A and 40 A in Figure 16a,d. The thermocouple collection does not respond promptly to the temperature fluctuations under the second-level power cycling. Overall, the thermocouple temperature is continuously increasing. The thermocouple temperature collected by Sensor1 is close to the temperature obtained by the IR camera. Sensor2 shows the temperature of $T_{middle}$ and analyzes heat diffusion of the chip horizontally.

**Figure 17:**
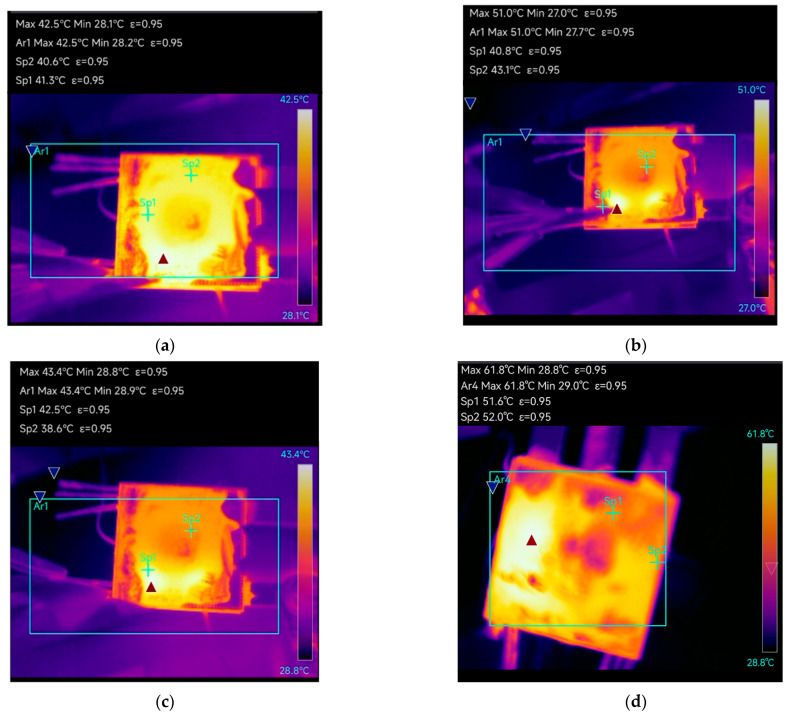
Low-side thermal image from IR camera. (**a**) ${PC}_{sec}$-30 A; (**b**) ${PC}_{sec}$-40 A; (**c**) ${PC}_{min}$-30 A; (**d**) ${PC}_{min}$-40 A.

The thermocouple data acquired during the turn-on state of the upper bridge arm, as illustrated in Figure 14, indicate that the ${PC}_{sec}$ thermal behavior can be captured by monitoring the temperature evolution on the DBC surface with an IR camera, as exemplified by the ${PC}_{sec}$ temperature distribution at 30 A and 40 A shown in Figure 16a,c and Figure 17a,c. Figure 16 and Figure 17 show the high-side and low-side thermal image from IR camera, respectively. Under second-level power cycling, the thermocouple fails to respond in a sufficiently prompt manner to the second-level temperature fluctuations, resulting in a monotonically increasing trend in the thermocouple-recorded temperature overall. A similar monotonically rising trend is also observed in the minute-level thermocouple temperature measurements. With increasing temperature, the values recorded by Sensor 1 closely approximate those obtained from the IR camera. Sensor 2 and Sensor 4 measure the temperature $T_{middle}$ and are employed to analyze the lateral heat diffusion from the chip.

The Temperature measurements were repeated three times under identical conditions to evaluate repeatability. The repeatability test was conducted after the module temperature had cooled down to room temperature following the completion of the specified power cycles. The results are reported as mean values and deviations in Table 5 and Table 6. Table 5 and Table 6 present the simulation and experimental results for the high side and low side. For each experimental case, the temperature difference between the simulated value and the measured value was calculated based on the maximum temperature across all data points. Specifically, ${\Delta T}_{3}$ represents the temperature difference between the simulated junction temperature and the actual measured junction temperature obtained by the IR camera. ${\Delta T}_{1}$ and ${\Delta T}_{2}$ denote the temperature differences between the simulated junction temperature and the simulated maximum temperature of the connected spacer 1 and spacer 2, respectively. These differences increase with chip power loss. ${\Delta T}_{6}$ and ${\Delta T}_{9}$ correspond to the temperature differences between the IR camera–measured junction temperature and the thermocouple-measured temperature placed on spacer 1 and spacer 2, respectively. The experimental results for these differences are in close agreement with the simulation results. ${\Delta T}_{4}$ and ${\Delta T}_{7}$ represent the simulated temperature differences across spacer 1 and spacer 2, respectively. ${\Delta T}_{5}$ and ${\Delta T}_{8}$ correspond to the experimentally measured temperature differences via thermocouples on spacer 1 and spacer 2, respectively. By comparing ${\Delta T}_{4}$ with ${\Delta T}_{5}$ and ${\Delta T}_{7}$ with ${\Delta T}_{8}$, the discrepancies between simulation and experiment for each spacer are evaluated. The results indicate that the simulated temperature differences are generally larger than the experimentally measured ones. These discrepancies are primarily attributed to factors such as the placement accuracy of thermocouples, the thermal conduction speed of the copper spacers, and the influence of forced air cooling on the thermal uniformity of the module. In some cases, these factors may cause certain measured temperatures to exceed the simulated junction temperature.

(19) $${\Delta T}_{1}=T_{j\_sim}-T_{spacer1\_sim}$$

(20) $${\Delta T}_{2}=T_{j\_sim}-T_{spacer2\_sim}$$

(21) $${\Delta T}_{3}=T_{IR}-T_{j\_sim}$$

(22) $${\Delta T}_{4}=T_{spacer1\_sim}-T_{spacer1\_mid\_sim}$$

(23) $${\Delta T}_{5}=T_{Sensor1}-T_{Sensor2}$$

(24) $${\Delta T}_{6}=T_{IR}-T_{Sensor1}$$

(25) $${\Delta T}_{7}=T_{spacer2\_sim}-T_{spacer2\_mid\_sim}$$

(26) $${\Delta T}_{8}=T_{Sensor3}-T_{Sensor4}$$

(27) $${\Delta T}_{9}=T_{IR}-T_{Sensor3}$$

where $T_{j\_sim}$ is the simulated maximum $T_{j}$ of the power device; $T_{spacer1\_sim}$ and $T_{spacer2\_sim}$ are the simulated maximum temperatures of spacer 1 and spacer 2, respectively; $T_{spacer1\_mid\_sim}$ and $T_{spacer2\_mid\_sim}$ are the simulated minimum temperatures of spacer 1 and spacer 2, respectively; $T_{IR}$ is the temperature measured by the IR camera; $T_{Sensor1}$, $T_{Sensor2}$, $T_{Sensor3}$ and $T_{Sensor4}$ are the measure result of Sensor1, Sensor2, Sensor3 and Sensor4, respectively; ${\Delta T}_{1}$–${\Delta T}_{9}$ are the temperature differences defined in Equations (19)–(27), respectively, all expressed in °C.

To show the accuracy of the performance between simulation and experiment. Table 7 provides the detailed data error from simulation and experiment. These results demonstrate excellent agreement between simulation and experiment. The root mean square error (RMSE) values of the high side and low side indicate that the model predictions deviate from the experimental measurements by more than the mean absolute error (MAE). The correlation coefficient (R^2^) values above 0.90 confirm that the variability in the experimental data is explained by the simulation model, validating the effectiveness of the proposed thermal modeling and thermal sensing approach.

(28) $$RMSE=\sqrt{\frac{1}{n}\sum_{i=1}^{n}{\left(y_{i}-{\hat{y}}_{i}\right)}^{2}}$$

(29) $$MAE=\frac{1}{n}\sum_{i=1}^{n}\left|y_{i}-{\hat{y}}_{i}\right|$$

(30) $$R^{2}=1-\frac{\sum_{i=1}^{n}{\left(y_{i}-{\hat{y}}_{i}\right)}^{2}}{\sum_{i=1}^{n}{\left(y_{i}-\bar{y}\right)}^{2}}$$

where $y_{i}$ is the measured temperature, ${\hat{y}}_{i}$ is the simulated temperature, and $\bar{y}$ is the mean of the measured temperature.

The experimental validation in this work employs K-type thermocouples as the sensing elements for the copper spacer temperature. These thermocouples are used solely for prototype characterization and thermal model calibration, enabling accurate quantification of the temperature difference.

Although the IR thermography provides a convenient reference for the maximum temperature distribution, several uncertainty sources must be considered. First, the IR camera is sensitive to the surface emissivity, which may vary with the black coating thickness and aging. Second, the finite spatial resolution and pixel size cause spatial averaging, especially near sharp temperature gradients, potentially leading to an underestimation of the maximum temperature. Third, the absolute temperature accuracy is limited by the camera calibration procedure and the accuracy of the reference thermocouple used for calibration. In addition, the thermocouples embedded in the copper spacers are affected by the exact sensor placement, the thermal contact quality, and the local thermal contact resistance. As a result, the reported temperature differences of approximately 0.5–2 °C between the spacer measurements and the IR-based reference should be interpreted within this combined measurement uncertainty, rather than as an absolute bound on the junction temperature error.

## 5. Conclusions and Future Perspectives

In this paper, a novel 3D DSC SiC power module utilizing vertical chip stacking and custom copper spacers was proposed and systematically evaluated. Compared to traditional SSC architectures, the introduced vertically symmetrical layout features an isothermal plane along the vertical direction of chips, which brings the junction temperature close to the copper spacer in the same vertical direction. The custom-designed copper spacers not only effectively reduce the overall thermal resistance but also achieve superior thermal balance between the top and bottom DBC during operation. Supported by the established transient thermal model, the custom-designed copper spacers serve dual functions as both vertical electrical interconnection and highly sensitive thermal proxies. This structural innovation directly simplifies the complexity of conventional junction temperature measurement. Through comprehensive power cycling simulations and experimental validations, it is demonstrated that the temperature deviation between the spacer measurement point and the IR camera reference temperature at the chip location is typically within approximately 0.5 °C to 2 °C. under the tested conditions, indicating close thermal coupling and supporting the feasibility of using the spacers as embedded sensing proxies. Finally, the proposed 3D chip stacking structure and embedded sensing methodology provide a reliable solution for the real-time condition monitoring and active thermal management of high-performance SiC modules.

Future research will focus on the structural optimization of the spacer and the evaluation of the module’s long-term operational reliability under rigorous thermo-mechanical stress. The proposed DSC structure is designed based on the SSC structure layout. Limited by the temperature rating of thermocouple adhesive, this study adopts 30 A and 40 A DC current to test the temperature distribution of the proposed module. The proposed model can incorporate practical integrated thermal sensing technology, which can be combined with AI technology to analyze the relationship between the spacer temperature and junction temperature. High thermal performance design for power modules, such as forced-air cooling, liquid cooling, using heat pipes, microchannel and direct substrate, can be applied to the proposed package design to further analyze the module’s current carrying capacity and enhance its power density under higher current conditions.

## Figures and Tables

**Table 1:** Dimensions of different layers inside the proposed 3D SiC module.

Layer	Material	Thickness (mm)
MOSFET die	Silicon Carbide	0.18
Die solder	Pb_92.5_Sn_5_Ag_2.5_	0.10
Upper copper	Copper	0.30
Ceramic	Aluminum nitride (AlN)	0.38
Lower copper	Copper	0.30
Bottom chip source connection solder1	Pb_92.5_Sn_5_Ag_2.5_	0.10
Spacer	Copper	3
Top chip source connection solder1	Sn_96.5_Ag_3_Cu_0.5_ (SAC305)	0.10

**Table 2:** Thermal properties of each material.

Material	Density ($kg/m^{3}$)	Specific Heat Capacity (J/(kg·°C))	Thermal Conductivity (W/(m·°C))
Pb_92.5_Sn_5_Ag_2.5_	7370	230	54
SAC305	11,020	250	40
SiC	3200	690	370
AlN	3320	780	200
Cu	8960	385	400

**Table 3:** Thermal difference in the chips in the same vertical position.

	${∆T}_{1\_3}$	$∆T_{2\_4}$	$∆T_{5\_7}$	$∆T_{6\_8}$
∆T (°C)	1.07	1.53	0.14	1.57

**Table 4:** Packaging material of the proposed DSC power module.

Part	Specifications
SiC MOSFET	1200 V/140 A, EPM3-1200-0017D, SiC MOSFET, Die dimension: 5 mm × 5 mm × 0.18 mm
DBC substrate	32.0 mm × 31.6 mm × 0.98 mm, Cu/AlN/Cu: 0.3 mm/0.38 mm/0.3 mm, copper plated with nickel and silver
Solder	Pb_92.5_Sn_5_Ag_2.5_ and SAC305 Preformed solder sheets according to the connection area
Spacer	Copper plated with nickel
Bonding Wire	5 mil Aluminum bonding wire to connect each chip gate pad and source pad
Power terminals	Copper plated with nickel
Signal terminals	Copper plated with nickel

**Table 5:** The experiment and simulation results of each case on the high side. Unit: °C.

Condition	$T_{j\_sim}$	$T_{spacer1\_sim}$	$T_{spacer1\_mid\_sim}$	$T_{IR}$	$T_{Sensor1}$	$T_{Sensor2}$	${\Delta T}_{1}$	${\Delta T}_{3}$	${\Delta T}_{4}$	${\Delta T}_{5}$	${\Delta T}_{6}$
${PC}_{sec}$-30 A	40.752	40.074	35.808	44.3	42.11	38.99	0.678	3.548	4.266	3.12	2.19
${PC}_{sec}$-40 A	52.034	50.772	42.834	52.3	50.80	46.74	1.262	0.266	7.938	4.06	1.5
${PC}_{min}$-30 A	45.382	44.500	40.652	45.1	44.57	41.64	0.882	−0.282	3.848	2.93	0.53
${PC}_{min}$-40 A	60.410	59.193	51.196	59.1	59.41	53.33	1.217	−1.31	7.997	6.08	−0.31

**Table 6:** The experiment and simulation results of each case on the low side. Unit: °C.

Condition	$T_{j\_sim}$	$T_{spacer2\_sim}$	$T_{spacer2\_mid\_sim}$	$T_{IR}$	$T_{Sensor3}$	$T_{Sensor4}$	Δ*T*_2_	Δ*T*_3_	Δ*T*_7_	Δ*T*_8_	Δ*T*_9_
${PC}_{sec}$-30 A	41.216	40.935	39.900	42.5	41.87	41.58	0.281	1.284	1.035	0.29	0.63
${PC}_{sec}$-40 A	52.389	52.064	50.101	51.0	49.16	48.01	0.325	−1.389	1.963	1.15	1.84
${PC}_{min}$-30 A	45.718	45.454	44.575	43.4	42.20	41.76	0.264	−2.318	0.879	0.44	1.2
${PC}_{min}$-40 A	61.042	60.555	58.704	61.7	61.57	59.40	0.487	0.658	1.851	2.17	0.13

**Table 7:** Quantitative metrics evaluation of the simulation model and experiment test.

Value	High Side *T_j_*	High Side *T_spacer_*	Low Side *T_j_*	Low Side *T_spacer_*
RMSE	1.90 °C	1.02 °C	1.53 °C	2.28 °C
MAE	1.35 °C	0.59 °C	1.41 °C	2.027 °C
$R^{2}$	0.93	0.98	0.96	0.91

## Data Availability

The original contributions presented in this study are included in the article. Further inquiries can be directed to the corresponding author.
